# ERK5 modulates IL-6 secretion and contributes to tumor-induced immune suppression

**DOI:** 10.1038/s41419-021-04257-8

**Published:** 2021-10-20

**Authors:** Kristina Riegel, Hajime Yurugi, Janine Schlöder, Helmut Jonuleit, Manuel Kaulich, Friederike Kirschner, Danielle Arnold-Schild, Stefan Tenzer, Hansjörg Schild, Krishnaraj Rajalingam

**Affiliations:** 1 Cell Biology Unit, University Medical Center Mainz, JGU-Mainz, Mainz, Germany; 2 Department of Dermatology, University Medical Center Mainz, JGU-Mainz, Mainz, Germany; 3 Gene Editing Group, Institute of Biochemistry II, Goethe University, Frankfurt, Germany; 4 Institute of Immunology, University Medical Center Mainz, JGU-Mainz, Mainz, Germany

**Keywords:** Cancer microenvironment, Extracellular signalling molecules

## Abstract

Tumors exhibit a variety of strategies to dampen antitumor immune responses. With an aim to identify factors that are secreted from tumor cells, we performed an unbiased mass spectrometry-based secretome analysis in lung cancer cells. Interleukin-6 (IL-6) has been identified as a prominent factor secreted by tumor cells and cancer-associated fibroblasts isolated from cancer patients. Incubation of dendritic cell (DC) cultures with tumor cell supernatants inhibited the production of IL-12p70 in DCs but not the surface expression of other activation markers which is reversed by treatment with IL-6 antibody. Defects in IL-12p70 production in the DCs inhibited the differentiation of Th1 but not Th2 and Th17 cells from naïve CD4^+^ T cells. We also demonstrate that the classical mitogen-activated protein kinase, ERK5/MAPK7, is required for IL-6 production in tumor cells. Inhibition of ERK5 activity or depletion of ERK5 prevented IL-6 production in tumor cells, which could be exploited for enhancing antitumor immune responses.

## Introduction

The tumor microenvironment has a profound impact on tumor growth and progression. Significant features of the tumor microenvironment are immune cells, stromal cells, blood vessels, and extracellular matrix [1]. This dynamic environment emerges during tumor progression when tumor cells elicit molecular, cellular, and physical changes [2] that often create an immune-suppressive milieu to favor tumor growth [1]. Such a pro-tumorigenic environment is characterized by the presence of regulatory cells like Tregs [3, 4], myeloid-derived suppressor cells (MDSCs) [5], modulated dendritic cells (DCs) [6], and alternatively-activated macrophages [7]. Immune suppression is further mediated through the secretion of factors such as TGF-β, IL-10, VEGF, and IL-6 by cancer cells and other cells present in the tumor [8].

IL-6 is considered one of the central players in tumor initiation, tumor growth, and metastasis by regulating fundamental processes like apoptosis, survival, proliferation, and angiogenesis [9]. Increased IL-6 serum levels have been demonstrated in several cancers including breast [10, 11] and lung cancer [12]. However, the critical role of IL-6 in tumorigenesis is even more underlined by the correlation of high levels of circulating IL-6 with a poor prognosis and lower survival of cancer patients [10, 12]. Not only cancer cells themselves are considered primary sources of IL-6, but also tumor-associated macrophages (TAMs), MDSCs, and cancer-associated fibroblasts (CAFs) [13, 14]. IL-6 does not only exhibit tumor cell-intrinsic activities but also tumor cell-extrinsic activities. For instance, it has been shown that STAT3, a downstream target of the IL-6 signaling pathway, induces the expression of target genes including VEGF and bFGF in TAMs and MDSCs, thereby contributing to tumor angiogenesis [15]. Because of the crucial role of IL-6 in cancer development, targeting the IL-6 pathway has been proposed to be a potent therapeutic approach [13]. In line with this, multiple studies were carried out to determine antitumor effects of monoclonal antibodies against IL-6, IL-6R, or sIL-6R or of selective inhibitors blocking the downstream signaling [13, 16–19].

Kinases in particular are popular targets for cancer therapeutics, as they constitute the major part of the “druggable genome” and deregulation in the kinome function is either directly or indirectly related to nearly 400 human diseases [20, 21]. Targeting the “oncogenic” kinases with ATP-competitive and non-competitive inhibitors in genetically defined human cancers has been very successful and has triggered tremendous interest in understanding the biology of the kinases to adroitly administer rational new generation kinase therapeutics. To date, the FDA has approved 62 kinase inhibitors for targeted therapeutics [22]. However, the function of the targeted kinases in immune regulation is only partially understood although the influence of targeted therapeutics on the immune cell function is critical for sustained tumor regression and enhanced patient survival. Thus, a thorough evaluation of the effect of targeted therapeutics on the immune system in preclinical animal models is crucial [23].

Inhibitors of the classical mitogen-activated protein kinase (MAPK) pathway encompassing RAF-MEK1/2-ERK1/2 give a prominent example of how targeted therapeutics can alter the antitumor immunity. It has been demonstrated that BRAF inhibition leads to increased infiltration of CD8^+^ T cells into the tumor but is also associated with an enhanced expression of the immunomodulatory molecule PD-L1 [24]. Further, already in 2006, it has been shown that the knockdown of BRAF V600E in melanoma cell lines resulted in a decrease of immunosuppressive factors like IL-10, VEGF, and IL-6 [25, 26].

Apart from the link between oncogenic MAPK pathway activation and suppression of antitumor immunity, there is also evidence that ERK5, the last discovered MAPK family member, is implicated in cancer-associated inflammation. For instance, inhibition of ERK5 in macrophages induces a transcriptional switch that blocked protumor macrophage polarization [27]. In the context of epidermal carcinogenesis, ERK5 is involved in controlling the expression of a subset of proinflammatory cytokines, and inhibition of ERK5 suppressed inflammation-driven tumorigenesis [28]. In this TPA-induced tumor model ERK5 was required to induce IL-1α, IL-1β, and COX-2, but not TNFα and IL-6 in epidermal keratinocytes [28]. In contrast, under stimulatory conditions it has been demonstrated that ERK5 contributes to the transduction of TLR2 signaling in human umbilical vein endothelial cells (HUVECs), the monocytic cell line Thp-1 as well as in human PBMCs and thus promotes the production of the cytokines IL-6 and IL-8 [29]. A follow-up study expanded the role of ERK5-dependent inflammation and showed that ERK5 regulates IL-6 secretion in HUVECs, HMVEC-lung cells, and monocytes downstream of diverse inflammatory mediators including the TLR2/TLR6-ligand fibroblast-stimulating ligand 1 (FSL-1), the TLR4-ligand lipopolysaccharide (LPS), IL-1β, and TNF-α [30].

Besides the involvement of ERK5 in inflammatory processes, ERK5 is known to regulate many cellular processes that are important for cancer cells [31]. Along this line, several studies have demonstrated a critical role of MEK5-ERK5 signaling in cancer cell proliferation and tumorigenesis. To name just a few examples, it has been shown that ERK5 positively affects the proliferation of prostate cancer cells in vitro and in vivo [32, 33], regulates the growth of chronic myeloid leukemia (CML)-patient-derived cells [34], and supports the proliferation and survival of multiple myeloma (MM) cells [35]. Further, various xenograft models, like a pancreatic tumor xenograft [36], a hepatocellular carcinoma xenograft [37], and a melanoma xenograft [38] underlined the relevant role of ERK5 in tumorigenesis since pharmacological inhibition of ERK5 resulted in tumor growth inhibition.

We performed an unbiased mass spectrometry-based secretome analysis and identified several factors including IL-6 being secreted from tumor cells and cancer-associated fibroblasts (CAFs). By secreting of IL-6, cancer cells inhibit IL-12p70 secretion from human moDCs, and the capacity of this modulated moDCs to induce a Th1 response is impaired. Interestingly, we uncover that ERK5 plays a critical role in the regulation of IL-6 secretion from several human lung cancer cell lines. Our study underlines how cancer cells can modify the immune response and highlights ERK5 as an interesting target in cancer therapy.

## Methods

### Cell culture

NCI-H226 (CRL-5826, ATCC), NCI-H2122 (CRL-5985, ATCC), and NCI-H1650 (CRL-5883, ATCC) were cultured in Roswell Park Memorial Institute (RPMI) medium (Cat. No. R8758, Gibco) containing 10% FCS and the A549 cells (DSMZ) in Dulbecco’s Modified Eagle Medium (DMEM) supplemented with 10% heat-inactivated FCS. The immortalized human lung epithelial cells (Saleb) and KRAS transformed SALEB (SaKRAS) were a kind gift from Dr. Scott Randell and were cultured in serum-free CnT-BM.1 medium with CnT-17.S supplement pack (CELLnTEC). These cells were originally selected with a triple antibiotic cocktail and characterized by RT-PCR confirmed expression of the genes used for immortalization and transformation [39]. The cancer-associated fibroblasts CCD-1065sk and CCD-1095sk were cultured in Dulbecco’s Modified Eagle Medium (DMEM) supplemented with 10% heat-inactivated FBS.

For experiments with the ERK5 inhibitors XMD 8-92 (Cat. No. 4132, TOCRIS) and XMD17-109 (concentration indicated in legends, Cat. No. A3942, ApexBio), cells were seeded in 12 well plates and treated once the cells reach near confluence (70-80%). NCI-H226, NCI-H1650, CCD-1065sk, and CCD-1095sk were pre-treated with the inhibitors for 4 h and NCI-H2122 for 2 h. After replacing the medium with fresh, inhibitor-containing RPMI medium NCI-H226, NCI-H1650, CCD-1065sk and CCD-1095sk were cultured for another 4 h and NCI-H2122 for another 5 h. Subsequently, supernatants were collected in order to study IL-6 secretion, while cells were lysed either in RIPA buffer or in TRIzol. Saleb and SaKRAS were pre-treated for 1 h with the ERK5 inhibitors XMD 8-92 (10 µM) or XMD17-109 (1 µM, Cat. No. A3942, ApexBio) in the presence of absence of IL-1β (10 ng/ml, Cat. No. 11340013, ImmunoTools). This was followed by 5 h incubation in the corresponding medium. In order to treat Saleb and SaKRAS with XMD 8-92 in the presence of Poly(I:C) (50 μg/ml, Cat. No. 27–4732–01, GE Healthcare), Poly(I:C) was transfected using Lipofectamine 2000 (Cat. No. 11668030, Thermo Fisher) followed by a 24 h treatment. Supernatants and cell lysates were subjected to further analysis.

To investigate the effect of the ERK5 inhibitor XMD 8-92 on cell viability, the listed cell lines were seeded in 96-well plates (NCI-H226 and NCI-H1650: 5 ×10^3^ cells per well, NCI-H2122: 15 × 10^3^ cells per well) and on the next day, the medium was changed to inhibitor-containing medium. Samples were analyzed after 6 h and 48 h.

In order to co-culture human monocytes during the differentiation to moDCs with the supernatant of NCI-H226, NCI-H2122, or A549, cancer cells were cultured until they reached confluence. After 2 more days, the supernatant was harvested and filtered through Minisart^®^ RC25 Syringe Filter (0.2 µm, Cat. No. 17764ACK, Sartorius). Optional, the supernatant was depleted of IL-6 by employing the capture antibody of the human IL-6 uncoated ELISA Kit (Cat. No. 88-7066). After rotating the supernatant overnight at 4 °C with the IL-6 specific antibody, antigen-antiboy complexes were precipitated by agarose-coupled protein A/G beads (Cat. No. 11-134-515-001 and 11- 243-233-001, Roche). After 3 h of rotation at 4 °C antigen-antibody complexes bound to the beads were removed by centrifugation for 5 min at 3000 rpm at 4 °C. The control supernatant was treated accordingly, but without the antibody.

### Secretome analysis and mass spectrometry

NCI-H226 were grown to 70% confluency in RPMI containing 10% FBS in T75 flasks and secreted proteins were isolated from cell culture supernatant using a click-chemistry-based approach similar to Eichelbaum et al. [40]. The medium was removed, the cells were washed twice with warm PBS and incubated for 30 min in 7 ml Starvation-Medium (RPMI-SILAC (Gibco ThermoFisher) with 10% FBS (Gibco by life technologies) without methionine, lysin, and arginine). After starvation, the medium was removed and labeling medium (RPMI-SILAC with 10% FBS, 0.8 mM lysine, 0.4 mM arginine, and 0.1 mM L-azidohomoalanine/AHA) was added. In addition, negative control with 0.2 mM methionine instead of AHA was carried along. After 18 h, the supernatant was collected, centrifuged for 5 min at 1000 × g and 4 °C to remove remaining cells and concentrated using Amicon Ultra-15 tubes (molecular mass cutoff 3000 Da, Millipore) to 0.25 ml. Three biological replicates were generated.

#### Enrichment and sample preparation

To isolate the newly synthesized, AHA-containing proteins from the media, Click-Chemistry-based enrichment was performed using the Jena Bioscience Click-Chemistry-Capture-Kit (Jena Bioscience) according to manufacturers protocol for both (AHA, control) supernatants and washed thoroughly to remove unspecifically bound proteins. For subsequent proteomic analysis by mass spectrometry, proteins bound on the beads were reduced, alkylated, and digested with trypsin. For digestion, beads were suspended in 50 µl digestion buffer (50 mM Tris, pH 8, 2 mM CaCl2 and 0.1% RapiGest), 0.5 μg trypsin (Promega) was added and incubated overnight at 37 °C. The peptide solution was collected, and the resin was washed with 50 μL 50 mM ammonium bicarbonate. Both solutions were combined and acidified with 5 μL 10% CF_3_COOH. Acidified samples were desalted on an Oasis HLB plate (Waters) according to manufacturer’s instructions. Desalted peptides were lyophilized and redissolved in 0.1% formic acid.

#### LC-MS analysis

A 2 µL of the reconstituted peptides were separated on an Ultimate 3000 nanoUPLC (Thermo Scientific) with 300 nL/min by a reversed-phase C18 column (HSS-T3 C18 1.8 μm, 75 μm × 250 mm, Waters Corporation) at 55 °C using a 90 min linear gradient from 5% Eluent A (0.1% TFA/3% DMSO/Water) to 35% Eluent B (0.1% TFA/3% DMSO/ACN) followed by ionization using a Nanospray Flex electrospray ionization source (Thermo Scientific). Mass-to-charge analysis of the eluting peptides was performed using an Orbitrap Exploris 480 (Thermo Scientific) in data-dependent acquisition (DDA) mode. Full scan MS1 spectra were collected over a range of 350–1600 m/z with a mass resolution of 60,000 ^@^ 200 m/z using an automatic gain control (AGC) target of 300%, maximum injection time was set to “Auto” and RF lens to 40%. The Top20 most intense peaks above the signal threshold of 2 × 10^4^, harboring a charge of 2–6, were selected within an isolation window of 1.4 Da as precursors for fragmentation using higher-energy collisional dissociation (HCD) with a normalized collision energy of 30. The resulting fragment ion m/z ratios were measured as MS2 spectra over an automatically selected m/z range with a mass resolution of 15,000^@^ 200 m/z, AGC target was set to “Standard” and maximum injection time to “Auto”.

#### Raw data processing and database search

Raw data processing and database searching was performed in MaxQuant (v1.6.17.0) [41] using the Andomeda Search Engine [42]. UniProtKB/SwissProt entries of the human reference proteomes (entries: 20,365) were used as a database for peptide and protein identification with a maximum allowed missed cleavages of two, maximum precursor, and fragment ion mass tolerance of 10 ppm and 0.02 Da respectively. Carbamidomethylation on cysteine (+57.021 Da) was set as only fixed modification. Oxidation on methionine (+15.995 Da) and N-terminal acetylation of proteins were set as variable modifications while allowing up to 3 dynamic modifications per peptide. Validation of the search results was performed using the Percolator algorithm [43] filtering for 1% False Discovery Rate (FDR).

### Chemical inhibitors

In addition to XMD 8-92, other MAPK inhibitors were screened to investigate their effects on IL-6 secretion. NCI-H226 cells were treated for 4 h with the MEK inhibitors CI-1040 (2 µM), Trametinib (2 µM) and U0126 (2 µM), the RAF inhibitors PLX-4032 (2 µM), PLX-4720 (2 µM) and the ERK5 inhibitor XMD 8-92 (2 µM). The medium was replaced by a fresh inhibitor-containing medium and after another 4 h of incubation supernatant was harvested in order to study IL-6 secretion.

### Cell proliferation assay

Cell proliferation and viability were determined using the Cell Proliferation Kit I (MTT, Cat. No 11465007001 ROCHE, SIGMA-ALDRICH) following the manufacturer’s instructions.

### EdU assay

NCI-H226, NCI-H2122, and NCI-H1650 cells were treated with XMD 8-92 (10 µM) in a 6-well cell culture plate for 24 h. EdU DNA synthesis assay was performed using Click-iT EdU pacific blue Imaging Kit (Thermo Fisher Scientific). The cells were incubated with EdU (5 µM) for 4 h. After staining the cells with the Fixable Viability Dye 780 (Cat. No. 65-0865-14, eBioscience) for 10 min at 4 °C, incorporated EdU was stained with the pacific blue ligand. The staining protocol was performed as described in the manufacture’s instruction and pacific blue-positive cells were analyzed by flow cytometer.

### Transfection of siRNA

Prior to siRNA transfection, NCI-H226 cells were cultured in a 12-well plate. siRNA was transfected at a final concentration of 60 nM using SaintRed as a transfection reagent. A scrambled control siRNA served as a negative control. The medium was changed 72 h after transfection. The supernatant was collected after 6 h incubation. siRNAs were purchased from Qiagen:

siControl(sense): 5’-UUCUCCGAACGUGUCACGU-3’ (Cat. No. 1027310)

siERK5 #1 (sense): 5’-GACCCACCUUUCAGCCUUA dTdT-3’ (Cat. No. S100606039)

siERK5 #2 (sense): 5’-CGAGAUCAUCGAGACCAUA dTdT -3’ (Cat. No. S100606046)

### CRISPR/Cas9 mediated knockout

CRISPR/Cas gRNA sequences targeting ERK5 were designed by Rule Set 2, as described previously (Doench et al., 2016). The top three scoring gRNAs (pick order sorted) were selected and individually cloned into pLenti-CRISPRv2 (Addgene plasmid #52961), following established protocols (Sanjana et al., 2014) [44].

For the production of lentiviral particles, the following packaging plasmids were used: pHDM-G (encoding VSV-G), pHDM Hgpm2 (encoding codon-optimized HIV gag-pol proteins), pHDM tat 1b (encoding HIV Tat1b protein), and pRC CMV-Rev1b (encoding HIV rev protein). Lentiviral particles coding for CRISPR_ERK5 and CRISPR control vector (pLentiCRISPRv2) were produced in 293 T cells that have been seeded in 6-Well plates. The lentiviral packaging plasmids (0.3 µg each) and 1.1 µg of the three lentiviral vectors containing the respective gRNAs were co-transfected in the presence of 21 µl of Lipofectamine2000 (Cat# 11668027, ThermoFisher Scientific). The viral particles were harvested after 48 h and sterile-filtered. NCI-H2122 were infected with lentiviral particles in the presence of 8 µg/ml of polybrene (Cat. No. sc-134220, Santa Cruz). Cells were then selected with 8 µg/ml puromycin (Cat# 0240.3, Carl Roth), until a stable knockout was achieved. DNA oligonucleotides encoding for ERK3 gRNA sequences were purchased from Sigma with the following sequences: ERK5-1-F 5’-CACCGttgaggacttccatgcacga-3’, ERK5-1-R 5’-AAACtcgtgcatggaagtcctcaaC-3’, ERK5-2-F 5‘-CACCGtgcctgcgtatactcgtgca-3‘, ERK5-2-R 5‘-AAACtgcacgagtatacgcaggcaC-3‘, ERK5-3-F 5’-CACCGaggctgcagagtcagatcaa-3’, ERK5-3-R 5’- AAACttgatctgactctgcagcctC-3’.

CRISPR/Cas gRNA sequences targeting ERK5 were designed by Rule Set 2 of Azimuth 2.0 as described previously (Doench et al., 2016). The top three scoring gRNAs (ERK5-1 ttgaggacttccatgcacga; ERK5-2 tgcctgcgtatactcgtgca; ERK5-3 aggctgcagagtcagatcaa) were selected and individually cloned into pLenti-CRISPRv2 (Addgene plasmid #52961), following established protocols (Sanjana et al., 2014) [44].

For the production of lentiviral particles, the following packaging plasmids were used: pHDM-G (encoding VSV-G), pHDM Hgpm2 (encoding codon-optimized HIV gag-pol proteins), pHDM tat 1b (encoding HIV Tat1b protein), and pRC CMV-Rev1b (encoding HIV rev protein). Lentiviral particles coding for CRISPR_ERK5 and CRISPR control vector (pLentiCRISPRv2) were produced in 293 T cells that have been seeded in 6-Well plates. The lentiviral packaging plasmids (0.3 µg each) and 1.1 µg of the three lentiviral vectors containing the respective gRNAs were co-transfected in the presence of 21 µl of Lipofectamine2000 (Cat# 11668027, ThermoFisher Scientific). The viral particles were harvested after 48 h and sterile filtered. NCI-H2122 were infected with lentiviral particles in the presence of 8 µg/ml of polybrene (Cat. No. sc-134220, Santa Cruz). Cells were then selected with 8 µg/ml puromycin (Cat# 0240.3, Carl Roth), until a stable knockout was achieved.

### Generation of human monocyte-derived dendritic cells (moDCs)

This study was conducted in accordance with the Declaration of Helsinki. Buffy coats were obtained from healthy volunteers at the University Medical Center Mainz with approval by the local ethical committee (Landesaerztekammer Rheinland-Pfalz). PBMCs were isolated from buffy coats following standard procedures [45] and 1.5 × 10^7^ PBMCs were seeded in pre-warmed RPMI (Cat. No. R8758, Gibco) containing 1% autologous plasma per well of a 6-well plate. The cells were allowed to adhere onto the plastic surface for 20 min at 37 °C/5% CO_2_. After removing non-adherent cells by washing with pre-warmed PBS, remaining adherent cells were cultured in RPMI supplemented with 10% FCS or were additionally treated with either recombinant human IL-6 (20 ng/ml, Immunotools) or with 20% of cancer cell supernatant (NCI-H226 or NCI-H2122). Differentiation into moDCs was carried out through the addition of 400 IU/ml human GM-CSF (Leukine, Sanofi) and 200 IU/ml recombinant human IL-4 (Cat. No. 11340045, Immunotools) as described before [46]. After 2 days, 1 ml medium was replaced by 1 ml of the corresponding RPMI medium (+10% FCS, + 200 IU/ml hIL-4, ±hIL-6 OR 20% cancer cell supernatant) with GM-CSF supplemented in a concentration of 800 IU/ml. Immature moDCs were harvested on day 5 of culture and 1–2 × 10^6^ cells were seeded per well of a new 6-well plate in RPMI supplemented with 10% FCS, 400 IU/ml GM-CSF, and 200 IU/ml IL-4 for maturation. The optional treatment with hIL-6 (20 ng/ml, Immunotools) or with 20% of cancer cell supernatant (NCI-H226 or NCI-H2122) was continued as well. Alternatively, the culture was carried out in presence of 20% NCI-H226 supernatant that was depleted of IL-6. Untreated and treated moDCs were stimulated with LPS (100 ng/ml) for 48 h and surface marker expression and cytokine secretion was characterized. Unstimulated moDCs served as controls. Optionally a control was included, in which cells were additionally treated with Tocilizumab (5 µg/ml, anti-IL-6R mAb) during the differentiation and stimulation. Alternatively, the culture was carried out in the presence of A549 supernatant (50%).

### Cytokine secretion

Cytokine secretion of NCI-H226, NCI-H2122, NCI-H1650, and human moDCs was measured by ELISA according to the manufacturer’s instructions (BD Bioscience). Collected supernatants were initially centrifuged for 10 min at 15000 rpm and 4 °C. Cancer cell lines were tested for their IL-6 secretion and moDCs for their IL-12p70, IL-8, IL-10, and TNF-α secretion. Alternatively, IL-12p70 secretion was determined by a Cytometric Bead Array (CBA) according to the manufacturer’s instructions.

### mRNA Isolation, cDNA synthesis and qPCR

RNA was isolated by TRIzol RNA extraction. Therefore, cells were washed with PBS and lysed in 1 ml TRIzol (Ambion/ThermoFisher). 200 µl chloroform was added to these samples and vortexed for 15 s followed by 2 min incubation at room temperature. Further, the samples were centrifuged at 14000 rpm for 15 min at 4 °C. The upper aqueous phase was transferred to a tube containing 0.5 ml isopropanol and incubated for 10 min at room temperature followed by another centrifugation step of 15 min at 14000 rpm at 4 °C. The pellet formed by the isolated RNA was washed once with 75% ethanol, air-dried, and resuspended in appropriate amount of ultrapure water. cDNA was synthesized from 1000 ng of the isolated RNA using the RevertAid First Strand cDNA Synthesis Kit (Cat. No. K1622, Thermo Scientific) and the supplied random hexamer primers.

All real-time PCR reactions were performed in triplicates on an iCycler (BioRad cxn96 or connect/Applied Biosystems Step One Plus) using EvaGreen (Cat. No. 27490, Axon). The mRNA levels of the housekeeping gene 18 S were used for normalization and relative expression levels were calculated as ∆∆Ct.

Real time PCR primers:

18 S: 5’-agaaacggctaccacatcca-3’ / 3’-caccagacttgccctcca-5’

IL-6: 5’-gcagaaaaaggcaaagaatc-3’ / 3’-ctacatttgccgaagagc-5’

IL-12A: 5’-atgagagttgcctaaattcc-3’ / 3’-cataaaagaggtctttctggag-5’

IL-12B: 5’-agaaagatagagtcttcacgg-3’ / 3’-aagatgagctatagtagcgg-5’

### Flow cytometry

In vitro generated and treated DCs were washed with PBS and stained for 30 min at 4 °C with the fluorescent-labeled antibodies listed in section “Antibodies”. To discriminate between live and dead cells, the cells were simultaneously treated with the Fixable Viability Dye 780 (Cat. No. 65-0865-14, eBioscience). After two washing steps with PBS, samples were acquired on a BD FACSCanto II, and data were analyzed with BD FACS Diva software (version 6.0) and FlowJo software. The mean fluorescence intensities (MFI) of independent experiments were quantified relatively to the corresponding controls.

For flow cytometric analysis of T cell differentiation, 0.5–1 × 10^6^ CD4^+^ T cells were treated with 100 µl of diluted (1:4) Fix/Perm buffer (Fixation/Permeabilization Concentrate, Cat. No. 00-5123 and Fixation/Permeabilization Diluent, Cat. No. 00-5223, eBioscience) for 45 min at 4 °C in the dark. Afterwards, fixed cells were washed with cold FACS buffer and incubated with diluted antibodies for transcription factors (see section “Antibodies”) for 30 min at 4 °C in the dark. Antibodies were diluted according to the manufacturer’s instructions in 1× Permeabilization buffer (Cat. No. 00-8333, eBioscience). After staining, cells were washed two times with 100 µl of 1× Permeabilization buffer and were resuspended in FACS buffer for flow cytometric analysis.

### T cell stimulation assays

The ability of DCs to induce T cell proliferation was investigated with a mixed lymphocyte reaction. As described in the methods, differentiation of monocytes to moDCs was performed in the presence or absence of IL-6 (20 ng/ml) or of 20% of cancer cell supernatant. After 5 days of differentiation, moDCs were stimulated in the corresponding medium with LPS for an additional 48 h. Unstimulated moDCs served as control. Cells were harvested, washed with PBS, and resuspended in X-VIVO-15 medium (Cat. No. BE04-418Q, Lonza) at a cell density of 0.5 ×10^6^ cells/ml. Freshly isolated T cells from allogeneic donors were used for proliferation assays. The enriched CD4^+^ T cells were washed with PBS and resuspended in X-VIVO-15 medium at a cell density of 1 ×10^6^ cells/ml. The mixed lymphocyte reactions were done in triplicates in a 96-well plate. The highest DC:T cell ratio was 1:2 (5 × 10^4^ DCs: 1 × 10^5^ CD4^+^ cells). A two-fold serial dilution was performed with DCs, which resulted in DC:T cell ratios ranging from 1:2–1:256. Controls containing DCs or CD4^+^ T cells alone were included. On day 4, ^3^H-thymidine was added to each well (37 kBq/well) and cells were cultured for an additional 16 h. DC-induced T cell proliferation was measured by ^3^H-thymidine incorporation using a liquid β-scintillation counter.

The differentiation of naïve T cells into effector cells induced by the treated moDCs was investigated. Naïve CD4^+^ T cells were purified from cord blood and cultured with the allogeneic moDCs. Alloreactive T cells were expanded and after 6 days a secondary stimulation with allogeneic DCs was performed. After 3 days, the differentiation of T cells was determined through the analysis of transcription factors.

### Antibodies

Antibodies used in this study recognize human antigens. Total ERK5 (rabbit monoclonal, Cat. No. 1719-1) was purchased from Epitomics. Phospho-Erk5 (Thr218/Tyr220) antibody (rabbit polyclonal, Cat. No. 3371) and antibodies against phosphorylated ERK1/2 at Tyr202/Tyr204 (rabbit polyclonal, Cat. No. 9101) and total ERK1/2 (rabbit polyclonal, Cat. No. 9102) were obtained from Cell signaling Technology. Anti-β actin (mouse monoclonal, HRP-conjugated, Cat. No. ab49900) and anti-β tubulin (rabbit polyclonal, HRP-conjugated, Cat. No. ab21058) were purchased from Abcam.

The following antibodies for flow cytometry were purchased from Biolegend: BV605 anti-human CD14 (Cat. No. 301833), BV605 mouse IgG2a (Cat. No. 400269), BV421 anti-human HLA-DR (Cat. No. 307635), BV421 mouse IgG2a (Cat. No. 400259), FITC anti-human CD80 (Cat. No. 305206), FITC mouse IgG1 (Cat. No. 400107), PerCP/Cy5.5 anti-human CD80 (Cat. No. 305231), PerCP/Cy5.5 mouse IgG1 (Cat. No. 400149). PE anti-human CD86 (Cat. No. 555665), PE mouse IgG2b (Cat. No. 555743), APC anti-human CD83 (Cat. No. 551073), APC mouse IgG1 (Cat. No. 555751) were purchased from BD Bioscience. Anti-human/mouse T-bet APC (Cat. No. 130-119-783, clone REA102) Miltenyi Biotec; Anti-human/mouse RORγT APC (Cat. No. 130-123-840, clone REA278) Miltenyi Biotec; PE anti-human GATA3 (Cat. No. 560074, clone L50-823) BD Bioscience; Alexa Fluor 647 anti-human Foxp3 (Cat. No. 32014, clone 259D), Biolegend

### SDS-PAGE and western blotting

Cell lysates were prepared in sodium dodecyl sulfate (SDS) sample buffer (0.125 M Tris-HCl, pH 6.8, 4% SDS, 10% glycerol, 10 mM DTT, and bromophenol blue) and loaded onto 7.5% polyacrylamide gels. The separated proteins were transferred onto nitrocellulose membranes (Cat. No. 10401296, Whatman Protran) using the wet/tank Blotting system from Bio-Rad. For immunoblot analysis, membranes were blocked with 3% BSA in PBS-T (PBS with 1% Triton X-100) for 1 h at room temperature. The incubation with primary antibodies was performed as suggested by the antibody providers. Horseradish peroxidase coupled secondary antibodies were then employed to visualize the antigen-antibody complexes by enhanced chemiluminescence (Cat. No. WBKLS0500, Millipore).

### Transcription factor filter plate assay

NCI-H226 were transfected with siControl or siERK5 as described above. After 48 h nuclear extracts were prepared by employing the Nuclear Extraction Kit (Cat# SK-0001, Signosis). 2 µg of the nuclear fraction were subjected to the filter plate assay. The activity of AP-1 (Cat. No. FA-0004, Signosis) and NF-κB (Cat. No. FA-0001, Signosis) were assessed according to the manufacturer’s instructions.

### Statistic analysis

P values were obtained by t-test in GraphPad Prism and if not stated otherwise *p* < 0.05 was considered as a significant difference. Statistical significance levels are annotated as *P < 0.05, **P < 0.01, ***P < 0.001.

## Results

**Fig. 1:**
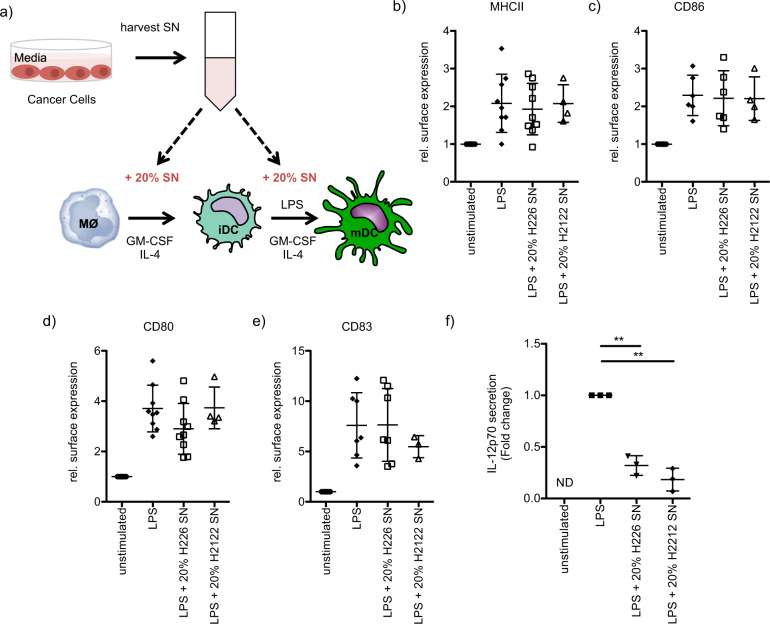
Impaired IL-12p70 secretion of human moDCs treated with cancer cell supernatant. **a** Schematic illustration of the experimental setup. Human moDCs were obtained through culturing monocytes with GM-CSF/IL-4 for 5 days. During the differentiation, cells were optionally treated with 20% of NCI-H226 supernatant (SN) or 20% of NCI-H2122 supernatant (SN). Immature moDCs (day 5 of culture) were stimulated for 48 h with LPS (100 ng/ml) in the corresponding medium. **b–e** Surface marker expression (**b**, MHCII, **c**, CD86, **d**, CD80 and **e**, CD83) was investigated by flow cytometry and the relative mean fluorescence of multiple independent experiments was quantified (mean fold change ± SD; Samples with 20% H226: MHCII: *n* = 9; CD86: *n* = 6; CD83: *n* = 7; CD80: *n* = 9; Samples with 20 % H2122 SN: MHCII: *n* = 4; CD86: *n* = 4; CD83: *n* = 3; CD80: *n* = 4). **f** moDCs were treated as described in a) and IL-12p70 secretion was studied by ELISA (mean fold change ± SD, *n* = 3, ND = not detected).

A central role of the tumor microenvironment has now also been recognized in lung cancer, the main cause of cancer-related mortality [47]. Indeed, the tumor microenvironment offers multiple targets such as VEGF and immune checkpoints for which novel anticancer agents can be developed [47, 48]. In our study, we were interested in evaluating how secreted factors from cancer cells influence the maturation and cytokine secretion of DCs, since these professional antigen-presenting cells are essential for a profound T cell-mediated cancer immunity [49, 50]. We investigated how culture supernatants of the human lung carcinoma cell lines NCI-H226, an NSCLC cell line with wild-type RAS, or NCI-H2122, a KRAS-mutated cell line, affect the maturation and cytokine secretion of LPS-stimulated moDCs (Fig. 1a). The addition of the culture supernatants to moDC cultures did not interfere with LPS-induced up-regulation of the surface markers MHCII, CD86, CD80, and CD83 (Fig. 1b–e and Supplementary Fig. 1). Interestingly, treating moDCs with the culture supernatants strongly inhibited LPS-induced IL-12p70 secretion (Fig. 1f). TNF-α levels were reduced as well when moDCs were stimulated in the presence of NCI-H226 culture supernatant, although the effect was less pronounced (Supplementary Fig. 2a). While IL-8 (Supplementary Fig. 2b) secretion was unaffected, IL-10 secretion was slightly enhanced after the addition of NCI-H226 culture supernatant to the moDC cultures (Supplementary Fig. 2c).

**Fig. 2:**
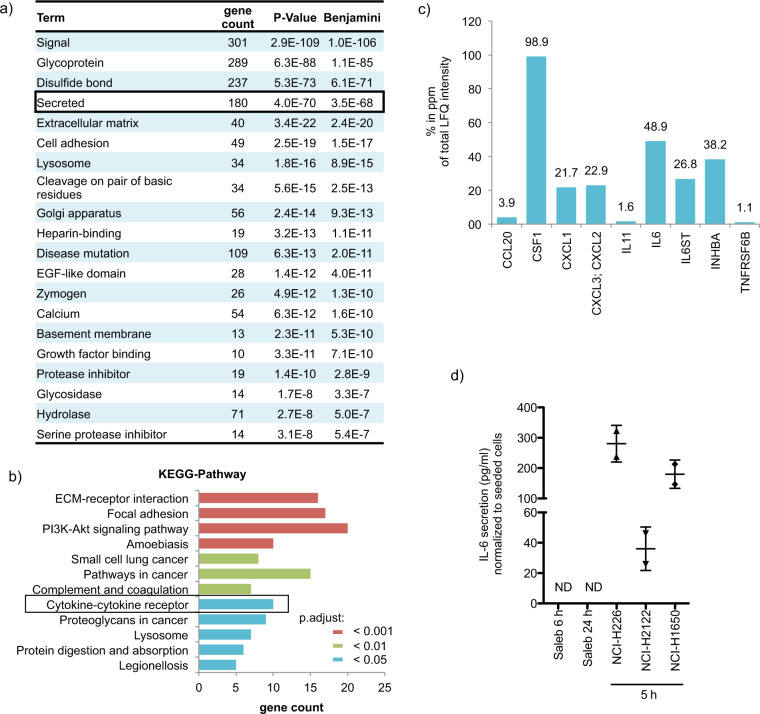
Identification of IL-6 among the secreted factors. **a** Mass-spectrometry based analysis was performed to identify secreted factors in the supernatant of NCI-H226 cells. The threshold of the LFQ intensities of identified proteins was set as log 2 ratio < −1. The identified secreted factors were classified using DAVID Bioinformatics Resources 6.8 with the settings “Functional_Categories: UP_Keywords”. Shown are the top 20 groups revealing among others the category “secreted”. **b** The 180 proteins of the in **a**, identified category “secreted” were further analyzed using DAVID Bioinformatics Resources 6.8 with the settings “KEGG pathways”. Only the pathways with Benjamin *p*-value< 0.05 are illustrated. **c** Displayed are the fa**c**tors of the in b, identified pathway “cytokine-cytokine receptor”. Shown are the respective percentages in ppm of the total LFQ intensities. **d** IL-6 secretion of NCI**-**H226, NCI-H2122, and NCI-H1650 was determined after 5 h incubation, while IL-6 secretion of Saleb cells was studied after 6 h and 24 h incubation (mean ± SD; *n* = 2).

Next, we wanted to identify the secreted factor(s) from the cancer cell lines causing the suppression of IL-12p70 secretion from stimulated moDCs. By combining mass spectrometry with AHA-labeling, we identified a large number of proteins in the cell culture supernatant of NCI-H226 cells, of which 425 proteins met our threshold criteria and had a log2 ratio < −1 compared to the unlabeled control. Analysis of these candidate proteins with the DAVID Bioinformatics Resources 6.8 allowed the functional categorization according to their UniProt Keywords (UP keywords) revealing the category “secreted”, among others (Fig. 2a and supplementary table 1). KEGG-pathway analysis of the 180 proteins assigned to the category “secreted” further specifies them as proteins being involved in pathways such as “focal adhesion”, “PI3K-Akt signaling pathway” as well as “cytokine-cytokine receptor” (Fig. 2b). Since cytokines play a pivotal role in the crosstalk between different cell types, we took a closer look at the proteins of the “cytokine-cytokine receptor” pathway. Among these secreted factors, CSF1, IL-6, and INHBA reveal the highest percentage of the total LFQ intensity (Fig. 2c). In the following, we concentrated on IL-6 due to studies reporting a role of IL-6 in DC maturation [51–53]. The production of IL-6 by NCI-H226 cells has been already demonstrated by Adachi et al., who showed that high levels of IL-6 promote cell growth in an autocrine manner [54]. In our own study, we confirmed the NCI-H226 cell line as IL-6 producer and revealed that NCI-H2122 cells also secrete IL-6 (Fig. 2d). NCI-H1650, another lung cancer cell line, harbors an EGFR-activating mutation and has been described as IL-6-secreting [55], which we could confirm in our study (Fig. 2d). Thus, IL-6 secretion from lung cancer cell lines is independent from the activating “driver” mutations. In contrast, IL-6 levels from Saleb cells, which are immortalized human lung epithelial cells, were undetectable even after 24 h of incubation (Fig. 2d).

**Fig. 3:**
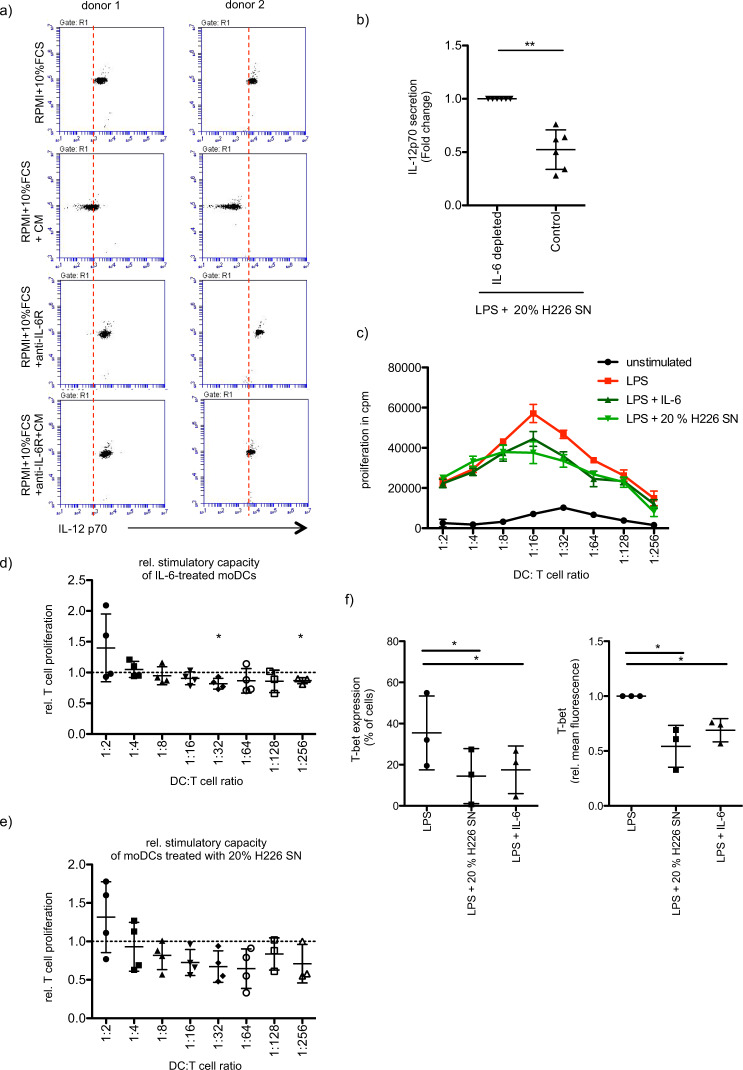
IL-6 secreted by cancer cells affects moDC-induced Th1 differentiation. **a** MoDCs were differentiated and stimulated (LPS, 100 ng/ml, 48 h) in the presence or absence of 20% of NCI-H226 supernatant (SN). Stimulation of moDCs was additionally carried out in the presence or absence of tocilizumab (5 µg/ml, anti-IL-6R mAb). IL-12p70 secretion was analyzed by CBA assay. Shown are 2 independent experiments. **b** Same as in a, but moDCs were differentiated and stimulated (LPS, 100 ng/ml, 48 h) in presence of IL-6 depleted- or control- supernatant (SN) of NCI-H226. The relative IL12-p70 secretion is presented (mean fold change ± SD; *n* = 6). **c** A MLR was performed with allogeneic CD4^+^ T cells and moDCs differentiated and stimulated (LPS, 100 ng/ml, 48 h) in the presence or absence of IL-6 (20 ng/ml) or of 20% of NCI-H226 supernatant (SN). T cell proliferation was determined via ^3^H-thymidine incorporation. Shown is a representative experiment. Error bars show the standard deviation of three technical replicates. **d** The induced T cell proliferation of IL-6-treated moDCs (20 ng/ml) (mean fold change ± SD; *n* = 4) and of **e**, moDCs treated with 20% of NCI-H226 supernatant (SN) was determined relative to the corresponding stimulated control (mean fold change ± SD; *n* = 4). **f** Naïve CD4^+^ T cells were expanded by stimulation with allogeneic moDCs, that have been differentiated and stimulated (LPS, 100 ng/ml, 48 h) in the presence or absence of IL-6 (20 ng/ml) or of 20% of NCI-H226 supernatant (SN). After restimulation of the CD4^+^ T cells with accordingly treated moDCs CD4^+^ T cells were stained for their T-bet expression. Shown is the percentage of the T-bet^+^ population (mean ± SD; *n* = 3) and the relative mean fluorescence of T-bet (mean fold change ± SD; *n* = 3).

Next, we addressed the question of whether IL-6 is the factor in the NCI-H226 supernatant that affects the LPS-induced secretion of IL-12p70 from moDCs. Indeed, the addition of tocilizumab (anti-IL-6R mAb) abrogated the inhibitory effect of NCI-H226 culture supernatant on IL-12p70 secretion from moDCs, suggesting a strong IL-6 dependency (Fig. 3a). This IL-6 dependency could be further confirmed in an IL-6 depletion experiment since moDCs cultured in the presence of IL-6-depleted NCI-H226 supernatant showed significantly stronger induction of IL-12p70 secretion compared to moDCs cultured in the NCI-H226 supernatant without prior IL-6 depletion (Fig. 3b). In line with this, the presence of IL-6 during moDC differentiation and maturation recapitulated our results on the secretion of IL-12p70, TNF-α, IL-8, and IL-10 seen after the addition of NCI-H226 or NCI-H2122 supernatants (Supplementary Fig. 3a-d). Despite high deviation, IL-12A and IL-12B mRNA levels are slightly reduced after treating LPS-stimulated moDCs with NCI-H226 culture supernatant or with IL-6 (Supplementary Fig. 3e,f) pointing to regulation on the transcriptional level. Additionally, we investigated how the supernatant of lung cancer cells that do not secrete IL-6 affects the LPS-induced maturation of moDCs. Therefore, we cultured moDCs in the presence of the supernatant of A549 cells. As with the supernatants of IL-6 producers, we did not observe any effects on the LPS-induced surface expression of moDCs cultured with A549 supernatant (Supplementary Fig. 4a). Interestingly, although there was a slight reduction in IL-12p70 secretion in the presence of A549 supernatant, this was far from the reduction observed in the presence of IL-6-containing supernatants (Supplementary Fig. 4b), thus again confirming IL-6 as an important factor to suppress IL-12p70 secretion from stimulated moDCs.

In order to evaluate the functionality of supernatant-treated moDCs co-culture experiments including allogeneic CD4^+^ T cells were performed. There was no significant impairment in the moDC-induced CD4^+^ T cell proliferation when LPS-stimulated moDCs were treated with either IL-6 **(**Fig. 3c, d**)** or with NCI-H226 culture supernatant (Fig. 3c, e). Since IL-12p70 is a Th1-polarizing cytokine [56], we tested, whether the polarization of naïve CD4^+^ T cells is affected, when NCI-H226 culture supernatant is added to the moDC culture. Indeed, the differentiation of naïve CD4^+^ T cell into T-bet^+^ CD4^+^ T cells was significantly impaired, when moDCs were cultured and stimulated in the presence of NCI-H226 culture supernatant (Fig. 3f). The addition of recombinant IL-6 caused a comparable reduction in T-bet^+^ CD4^+^ T cells (Fig. 3f). Analysis of the expression of GATA3, RORγt, and Foxp3 did not reveal clear effects on the differentiation of naïve CD4^+^ T cell into Th2, Th17, and Tregs (Supplementary Fig. 5). These data indicate that IL-6 secreted from different lung cancer cell lines can contribute to an impaired Th1 response through the inhibition of IL-12p70 secretion by moDCs.

**Fig. 4:**
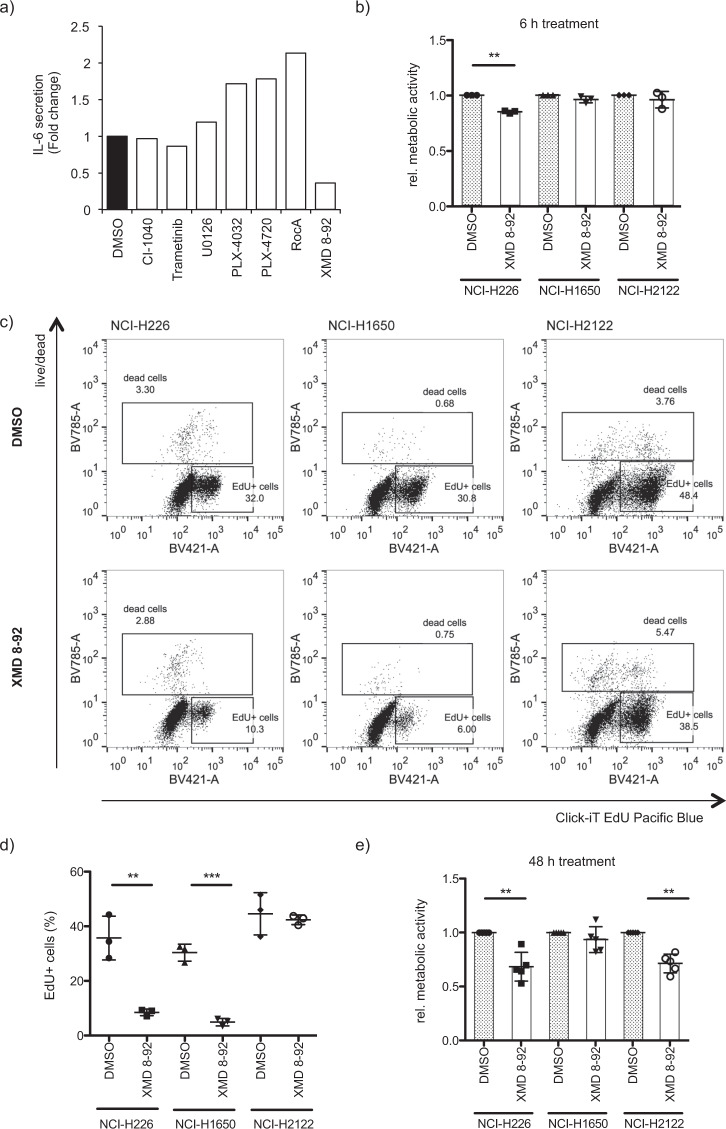
ERK5 inhibition negatively influences viability of cancer cells. **a** NCI-H226 cells were treated for 4 h with a panel of MAPK pathway inhibitors including the MEK inhibitors CI-1040 (2 µM), Trametinib (2 µM) and U0126 (2 µM), the RAF inhibitors PLX-4032 (2 µM), PLX-4720 (2 µM) and RocA (400 nM) and the ERK5 inhibitor XMD 8-92 (2 µM). The medium was replaced by a fresh inhibitor-containing medium and after another 4 h of incubation, the supernatant was harvested. IL-6 secretion was determined by ELISA (*n* = 1). **b** Metabolic activity of the cancer cell lines NCI-H226, NCI-H1650, and NCI-H2122 was measured by MTT assay after treating cells for 6 h with DMSO or XMD 8-92 (10 µM) (mean fold change ± SD; *n* = 3). **c** NCI-H226, NCI-H1650, and NCI-H2122 were treated for 24 h with DMSO or XMD 8-92 and EdU assay was performed to evaluate the amount of newly synthesized DNA by flow cytometry analysis following the manufacturer’s instructions. A representative experiment is shown illustrating the identification of EdU^+^ cells. **d** The amount of EdU^+^ cells was quantified (mean ± SD; *n* = 3). **e** Same as in b, but metabolic activity was measured after 48 h treatment (mean fold change ± SD; *n* = 4).

We were then interested in deciphering how IL-6 secretion is regulated in the NCI-H226 cell line. Therefore, we screened a panel of MAPK-inhibitors for their ability to modulate IL-6 secretion (Fig. 4a). Interestingly, the inhibitors blocking the RAF-MEK1/2-ERK1/2 pathway showed no or at most a promoting effect on IL-6 secretion, whereas the ERK5 inhibitor XMD 8-92 significantly reduced secretion. The inhibitory effect of XMD 8-92 on IL-6 secretion was not caused by cell death, because metabolic activity was largely unaffected at the time point when IL-6 secretion was reduced **(**Fig. 4b**)**. The antitumor effect of XMD 8-92 has been already demonstrated with a cervical xenograft model in immunodeficient mice and with a lung xenograft model in immunocompetent mice [57]. In line with this, we demonstrate, that XMD 8-92 treatment of NCI-H226 and NCI-H1650 cells significantly impaired the incorporation of EdU after 24 h of treatment indicating a striking reduction in the proliferation (Fig. 4c, d). In case of NCI-H2122 no difference in EdU incorporation was detected, although viability was significantly decreased after 48 h (Fig. 4e). Thus, our data show that XMD 8-92 has an inhibitory effect on the growth of the lung cancer cell lines used in this study.

**Fig. 5:**
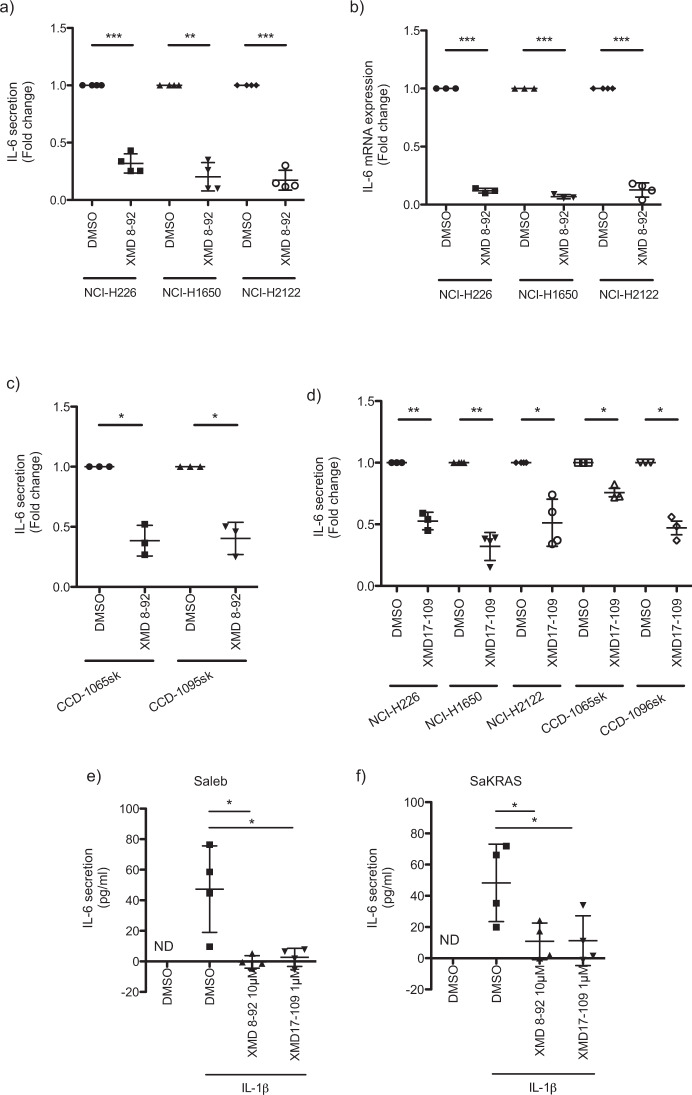
ERK5 regulates IL-6 secretion from various cancer cell lines. **a** The cancer cell lines NCI-H226 and NCI-H1650 were treated for 4 h and NCI-H2122 for 2 h with DMSO or XMD 8-92 (10 µM). After changing the medium to fresh inhibitor-containing medium, supernatant of NCI-H226 and NCI-H1650 was harvested after 4 h, and in the case of NCI-H2122 after 5 h of incubation. IL-6 secretion was studied by ELISA (mean fold change ± SD; *n* = 4) and **b**, IL-6 mRNA levels were determined by real-time PCR (mean fold change ± SD; NCI-H226/NCI-H1650: *n* = 3; NCI-H2122: *n* = 4). **c** Same as in (**a**), but with CCD-1065sk and CCD-1095sk fibroblasts (mean fold change ± SD; *n* = 3). **d** NCI-H226, NCI-H1650, CCD-1065sk, and CCD-1095sk fibroblasts were pre-treated with XMD 17-109 (NCI-H226, CCD-1065sk, and CCD-1095sk fibroblasts: 10 µM; NCI-H1650, NCI-H2122: 5 µM) for 4 h and NCI-H2122 for 2 h. After changing the medium to fresh inhibitor-containing medium, supernatant of NCI-H226, NCI-H1650, CCD-1065sk, and CCD-1095sk fibroblasts were harvested after 4 h, and in the case of NCI-H2122 after 5 h of incubation. IL-6 secretion was determined by ELISA (mean fold change ± SD; NCI-H226, CCD-1065sk and CCD-1095sk fibroblasts: *n* = 3; NCI-H1650, NCI-H2122: *n* = 4). **e** Saleb, and **f**, SaKRAS cells were pre-treated with DMSO, XMD 8-92 (10 µM) or XMD17-109 (1 µM) in presence or absence of IL-1β (10 ng/ml) for 1 h. The medium was replaced, and treatment was continued for 5 h. The supernatant was collected, and IL-6 secretion was determined by ELISA (mean ± SD; *n* = 4).

Regarding IL-6 our data suggest that independent of the driver mutation of the three lung cancer cell lines (NCI-H226, NCI-H2122, and NCI-H1650), treatment with XMD 8-92 significantly reduces its secretion (Fig. 5a). The decreased IL-6 secretion is probably caused by transcriptional regulation, as IL-6 mRNA levels were significantly diminished in all three cell lines after XMD 8-92 treatment (Fig. 5b). Since CAFs play a pivotal role in tumorigenesis through the secretion of several factors including IL-6 [58, 59], we tested whether IL-6 secretion is dependent on ERK5 in CAFs as well. Indeed, treatment with the ERK5 inhibitor XMD 8-92 (Fig. 5c) caused a significant reduction in IL-6 secretion from two different CAF lines. Next, we wanted to confirm the inhibitory effect on IL-6 secretion with a second ERK5 inhibitor. Indeed, XMD 17-109 significantly reduced IL-6 secretion from the three lung cancer cell lines NCI-H226, NCI-H2122, and NCI-H1650 as well as from the two CAFs lines **(**Fig. 5d**)**.

In Fig. 2d, we demonstrated that IL-6 levels of Saleb cells are undetectable. Stimulation of Saleb with IL-1β induces IL-6 secretion, which is blocked in the presence of the ERK5 inhibitors XMD 8-92 and XMD17-109 (Fig. 5e). This is independent of KRAS mutation, since KRAS-transformed SALEB cells (SaKRAS) recapitulate the observed ERK5-dependent induction of IL-6 (Fig. 5f). We have obtained comparable results when we stimulated Saleb or SaKRAS cells with Poly(I:C) (Supplementary Fig. 6a,b). Next, we were interested in whether the stimulation of Saleb cells not only induced IL-6 secretion but also ERK5 phosphorylation. Therefore, we monitored the activating ERK5 phosphorylation at different time points. Indeed, we observed a slight but reproducible induction in ERK5 phosphorylation that was highest after 1 h of stimulation and decreases thereafter **(**Supplementary Fig. 7a,b). In contrast, despite the high variability, our data suggested that ERK1/2 signaling was also activated and that activation was highest after only 15 min of stimulation. (Supplementary Fig. 7a, c). We obtained similar results with IL-1β-stimulated SaKRAS cells, although the highest ERK5 as well as ERK1/2 activation were reached after about 15 min (Supplementary Fig. 7d–f).

**Fig. 6:**
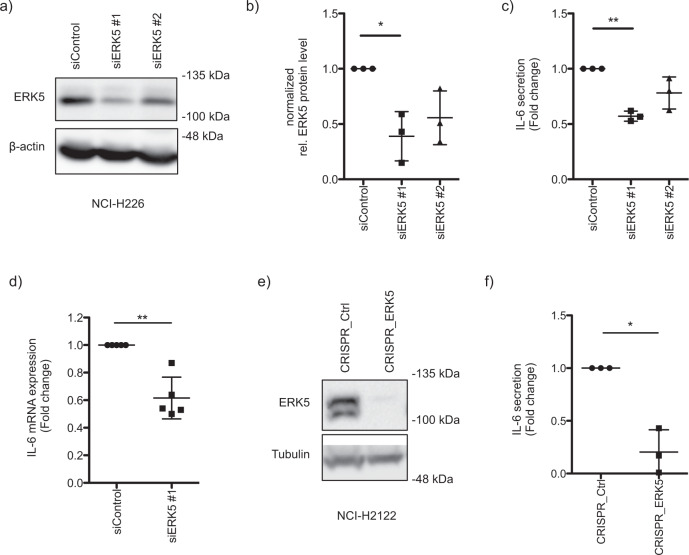
ERK5-specific effect on IL-6 secretion. **a** siRNAs were transfected to specifically knockdown ERK5 in NCI-H226 cells and the knockdown of ERK5 was confirmed by Western blot analysis. **b** The siRNA-mediated knockdown efficiency was quantified by normalizing the ERK5 levels to the corresponding tubulin or ponceauS staining (mean fold change ± SD; *n* = 3). **c** Same as in a, but after 72 h IL-6 secretion was studied by ELISA. Therefore, the medium was replaced by a fresh medium, and the supernatant was harvested after 6 h of incubation to determine IL-6 secretion (mean fold change ± SD; *n* = 3). **d** Same as in a, but IL-6 mRNA levels were determined by qPCR 48 h after siRNA transfection (mean fold change ± SD; *n* = 5). **e** CRISPR/Cas-mediated knockout of ERK5 was induced in NCI-H2122 cells and confirmed by Western blot analysis. **f** NCI-H2122 cells transfected with CRISPR_Control or CRISPR_ERK5 were seeded in 12-well plates. After cells have adhered overnight, the medium was changed to a fresh culture medium. The supernatant was harvested after 6 h of incubation and IL-6 secretion was studied by ELISA (mean fold change ± SD; *n* = 3).

To demonstrate the ERK5-specific effect on IL-6 secretion, loss-of-function studies were performed. Indeed, we confirmed that siRNA-induced knockdown of ERK5 in NCI-H226 cells (Fig. 6a, b) resulted in a significant reduction in IL-6 secretion (Fig. 6c). Due to reduced IL-6 mRNA levels after siRNA-mediated knockdown of ERK5, our data supported the hypothesis of transcriptional regulatory mechanisms (Fig. 6d). We additionally confirmed the ERK5-specific inhibition of IL-6 secretion by inducing a CRISPR-mediated knockout of ERK5 in NCI-H2122 (Fig. 6e, f).

**Fig. 7:**
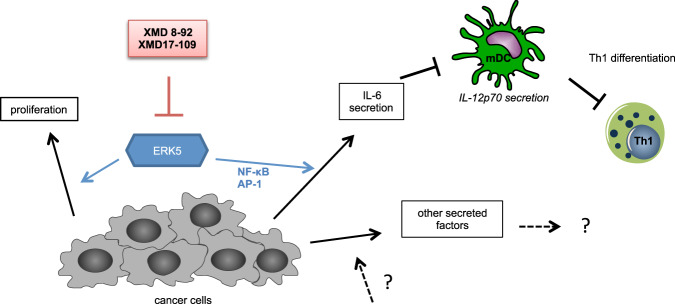
ERK5 inhibitors impair cancer cell viability and inhibit IL-6 secretion. Schematic illustration of how XMD 8-92 affects a subset of cancer cells. In brief, ERK5 modulates IL-6 secretion of cancer cells and thereby also tumor-induced immune suppression since IL-6 secreted by cancer cells impairs IL-12p70 secretion of moDCs that in turn negatively affects Th1 differentiation.

All together, our study proposes ERK5 as a promising target for cancer therapies in light of the fact that ERK5 modulated not only the cancer cell viability but also the IL-6 production. IL-6 secreted by cancer cells impaired the IL-12p70 secretion from DCs, which in turn had an effect on the differentiation of CD4^+^ T cells (Fig. 7).

## Discussion

Because of the clinical success of kinase inhibitors in genetically defined human cancers extensive studies were carried out in order to characterize the contribution of oncogenic kinases to tumorigenesis. Although targeted therapeutics resulted in profound clinical responses, they were often not durable. Another approach in cancer treatment is the development of immune-based therapeutics in order to promote antitumor immunity. While those therapeutics accomplished more durable clinical responses, the overall response rate was limited [23]. However, the combination of targeted therapy and immunotherapy provides a promising strategy since the advantages and limitations of these regimens seem to be complementary [60]. In order to design effective combination strategies, it is warranted to evaluate in which way targeted therapeutics influence the antitumor immune response.

ERK5 has been already proposed as a promising target in cancer therapy, because experiments with preclinical models provided evidence that ERK5 inhibitory strategies are beneficial for cancer treatment [31]. Furthermore, treatment with XMD 8-92 was described to lead to tumor growth inhibition in a lung cancer xenograft model [57]. This is in line with our in vitro data, demonstrating a significant reduction of the proliferation/viability of the tested lung cancer cell lines (NCI-H226, NCI-H2122, and NCI-H1650) after ERK5 inhibition **(**Fig. 4**)**. The importance of ERK5 in lung cancer is further underlined by the observation that human lung tumor samples had enhanced MEK5 and ERK5 expression levels, which were significantly correlated with poor overall prognosis [61].

In the present study, we identified ERK5 as an important regulator of IL-6 in several lung cancer cell lines (Figs. 5, 6). We demonstrated transcriptional regulation of IL-6 by ERK5, but further studies are needed to provide more mechanistic insights. Therefore, it has to be addressed whether ERK5 modulates IL-6 levels by influencing the IL-6 promoter activity and what regulatory mechanisms underlie this phenotype. Previous studies have already described cis-regulatory elements in the human IL-6 gene. These functional elements include an AP-1 (activator protein-1) consensus site [62, 63], a cAMP response element (CRE) [64], a nuclear factor (NF)-IL6 binding site [65, 66] and an NF-κB-responsive element [67] (Supplementary Fig. 8). In fact, we have tested the influence of a siRNA-mediated knockdown of ERK5 in NCI-H226 on the activity of the aforementioned transcription factors by employing a filter plate assay (Supplementary Fig. 8). Here, we observed that in ERK5-knockdown cells the activity of AP-1 was slightly impaired, while the negative impact on NF-κB activity was more pronounced. In contrast, CREB and C/EBP activities were largely unaffected (data not shown). As suggested by Faggioli *et al*., there is a strong synergism among NF-κB, C/EBP, and AP-1, which is important for the regulation of the IL-6 promoter [68]. Although only the NF-κB binding site is required for IL-6 promoter activation, NF-κB alone is not sufficient to induce the IL-6 promoter upon IL-1 or TNF-α stimulation because interactions with AP-1 and/or C/EBP are also required [68]. However, it has been shown that the colon cancer cell line HTM-29 cannot secrete IL-6 upon IL-1β- or TNF-α-stimulation, because these cytokines do not lead to NF-κB activation here [69]. Interestingly, the regulatory role of ERK5 in modulating the activity of especially NF-κB has been already described. For instance, in colon cancer, it has been shown that pharmacological inhibition of ERK5 by employing XMD 8-92 inhibitor resulted in decreased IL-8 expression and NF-κB transcriptional activity [70], and also in Jurkat cells it was observed that ERK5 controlled the nuclear localization of p65, a subunit of NF-κB [71]. Further, only the co-expression of ERK5 with a constitutively active mutant of the upstream kinase MEK5 activated an NF-κB reporter gene in Jurkat cells. Neither vector alone induced NF-κB activation [71] suggesting that the ERK5 dependent activation of NF-κB is kinase-dependent. Since our filter plate assays suggest that ERK5 inhibition interferes with the activation of the transcription factors relevant for IL-6 promoter activation, we hypothesize that the reduced IL-6 levels might be the result of an insufficient induction of the IL-6 promoter activity.

The regulatory role of ERK5 in IL-6 production is not unique to lung cancer cells, since we further confirmed ERK5-dependent IL-6 secretion in CAFs (Fig. 5). Additionally, it has been demonstrated that ERK5 contributes to the transduction of TLR2 signaling in Thp-1 cell lines as well as in human PBMCs and thus promotes the production of the cytokines IL-6 and IL-8 [29].

IL-6 is a pleiotropic cytokine that balances pro-and anti-inflammatory conditions. But IL-6 has been also described as a driver of tumorigenesis [9] and high levels of circulating IL-6 concentrations are correlated with a poor prognosis and lower survival of cancer patients [10, 12]. In addition to its tumor cell-intrinsic activities, tumor cell-extrinsic activities like the promotion of tumor angiogenesis are described as well [15]. In our study, we demonstrate that IL-6 could additionally alter the antitumor immune response through modulating DCs, since IL-6 secreted from lung cancer cell lines inhibited the IL-12p70 production from LPS-stimulated moDCs (Fig. 1). As expected, the ability of the IL-6- or cancer supernatant treated moDCs to induce the differentiation of naïve CD4^+^ T cell into T-bet^+^ CD4^+^ T cell was impaired (Fig. 3). These observations are in line with a recent study by Ohno *et al*., showing that the interferon (IFN)-γ production of CD4^+^ T cell co-cultured with IL-6-conditioned moDCs was attenuated [52]. The in vivo significance of IL-6 modulating DC function was then shown in a further study investigating the immune status of CT26 tumor-bearing mice that are deficient for IL-6. Higher numbers of mature DCs, CD4^+^ T cells, and CD8^+^ T cells were present in tumor sites of IL-6 deficient mice compared with wild-type mice, thus underlining the pivotal role of IL-6 in tumorigenesis [72].

We had identified IL-6 in an unbiased mass spectrometry-based secretome analysis (Fig. 2) and concentrated on this factor due to already reported functions of IL-6 in DC maturation [51–53]. In addition to IL-6, other factors were detected that might contribute to an altered antitumor immune response. For instance, among the other factors attributed to the KEGG pathway “cytokine-cytokine recptor”, chemokines were identified which have an influence on the recruitment of immune cells to the tumor. One of the identified chemokines is CXCL1, which has been described to promote lung cancer growth through the recruitment of tumor-associated neutrophils [73]. In addition, CXCL3 and CXCL2 have been identified in the screen, which can also function as chemoattractants for neutrophils [74–76]. Intriguingly, CXCL1, CXCL2, and CXCL3 are members of the angiogenic CXC chemokine family promoting endothelial cell chemotaxis thus playing a pivotal role in angiogenesis [77]. In line with this, the secretome anaylsis revealed that VEGFA, an important factor in mediating angiogenesis [78], is secreted by NCI-H226 cells as well. In contrast, the identified chemokine CCL20 represents a factor that can affect cancer cells in an autocrine and paracrine manner promoting cancer progression by enhancing cancer cell migration and proliferation [79].

In our study, we highlight ERK5 as a promising target for cancer therapies since ERK5 modulates not only the cancer cell viability but also the IL-6 production, which is involved in the regulation of type-1 immunity probably through the downregulation of IL-12p70 secretion from DCs (Fig. 7). Further studies are required to provide more mechanistic details on how ERK5 contributes to IL-6 secretion.

## Supplementary information

Supporting materials

Figure S1

Figure S2

Figure S3

Figure S4

Figure S5

Figure S6

Figure S7

Figure S8

Supplementary table

## Data Availability

All data and materials which are not commercially available employed in the study are available upon request from the authors.
