# Supplementary material for: ERK5 modulates IL-6 secretion and contributes to tumor-induced immune suppression
Source: Cell Death Dis. 2021 Oct 20;12(11):969. doi: 10.1038/s41419-021-04257-8 (PMC8528934; doi:10.1038/s41419-021-04257-8)
Supplement: Supplementary file 1 — Supporting materials [file 41419_2021_4257_MOESM1_ESM.docx]

**Supplementary Fig. 1 Gating strategy of human moDCs.** Surface markers of moDCs (MHCII, CD86, CD80 and CD83), which were differentiated and stimulated (LPS, 100 ng/ml, 48h) in presence or absence of IL-6 (20 ng/ml), 20% of NCI-H226 supernatant (SN) or of 20% of NCI-H2122 supernatant (SN) were analyzed by flow cytometry. Shown is a representative experiment illustrating the gating scheme.

**Supplementary Fig. 2 Influence of NCI-H226 supernatant on cytokine secretion of moDCs. a,** To obtain human moDCs, monocytes isolated from buffy coats were cultured for 5 days in the presence of GM-CSF/IL-4. During the differentiation, cells were optionally treated with 20% of NCI-H226 supernatant (SN). Immature moDCs (day 5 of culture) were stimulated for 48 h with LPS (100 ng/ml) in the corresponding medium and secretion of TNF- α (mean fold change ± SD, n = 5), **b**, IL-8 (mean ± SD, n = 3) and **c**, IL-10 (mean ± SD, n = 4) was determined by ELISA (ND = not detected).

**Supplementary Fig. 3 Effect of secreted IL-6 from cancer cells on IL-12A and IL-12B mRNA levels of human moDCs. a,** MoDCs were differentiated and stimulated (LPS, 100 ng/ml, 48h) in presence or absence of IL-6 (20 ng/ml) and cytokine secretion of IL12-p70 (mean ± SD, n = 3), **b,** TNF- α (mean fold change ± SD, n = 5), **c**, IL-8 (mean ± SD, n = 3) and **d**, IL-10 (mean ± SD, n = 4) was studied by ELISA (ND = not detected). **e,** MoDCs were differentiated and stimulated (LPS, 100 ng/ml, 48h) in presence or absence of IL-6 (20 ng/ml), 20% of NCI-H226 supernatant (SN) or 20% of NCI-H2122 supernatant (SN) and IL-12A and **f,** IL-12B mRNA levels were determined by real time PCR (mean fold change ± SD, n = 3).

**Supplementary Fig. 4 Effect of A549 supernatant on human maturation. a**, Same as in Fig. 1, but differentiation and maturation of moDCs was carried out in presence of 50% of A549 supernatant. Surface marker expression (MHCII, CD86, CD80 and CD83) was investigated by flow cytometry and the relative mean fluorescence of four independent experiments was quantified (mean fold change ± SD; in case of CD80: n = 2)). **b**, moDCs were treated as described in a) and IL-12p70 secretion was studied by ELISA (mean fold change ± SD, n = 3, ND = not detected).

**Supplementary Fig. 5 Analysis of transcription factors of naïve CD4^+^ T cells after stimulation with moDCs. a,** According to Figure 4d, naïve CD4^+^ T cells were expanded and restimulated with moDCs, which were differentiated and stimulated (LPS, 100 ng/ml, 48h) in presence or absence of IL-6 (20 ng/ml) or of 20% of NCI-H226 supernatant (SN). Expression of GATA3, **b,** RORγt and **c,** FoxP3 was analyzed by flow cytometry. Shown is the relative mean fluorescence of two independent experiments (mean fold change ± SD).

**Supplementary Fig. 6 ERK5 inhibited IL-6 secretion in Poly(I:C) stimulated Saleb and SaKRAS cells. a,** Saleb and **b,** SaKRAS cells were pre-treated with DMSO or XMD 8-92 (10 µM) for 1 h. After replacing the medium with fresh inhibitor-containing medium, Poly(I:C) was transfected and stimulation was continued for 24 h. Supernatant was collected, and IL-6 secretion was determined by ELISA (mean ± SD, Saleb: n = 3; SaKRAS: n = 4).

**Supplementary Fig. 7 ERK5 acitvation upon IL-1β stimulation a**, Saleb were stimulated with IL-1β (10 ng/ml) and activating phosphorylation of ERK5 (Thr218/Tyr220) and ERK1/2 (Tyr202/Tyr204) were monitored by Western blot (representative experiment shown). **b,** ERK5- and **c,** ERK1/2 phosphorylation were quantified in relation to their total levels (mean fold change ± SD, n = 5). **d-f,** Same as in a-c, but with SaKRAS cells (mean fold change ± SD, n = 4).

**Supplementary Fig. 8 Influence of ERK5 on transcription factors of the IL-6 promoter. a,** Schematic illustration of the IL-6 promoter and relevant transcription factor binding sites (adapted from (1)). **b,** NCI-H226 were transfected with siControl or siERK5 and after 48 h, activity of the transcription factors AP-1 (mean fold change ± SD, n = 2) and **c,** NF-κB (mean fold change ± SD, n = 3) were measured by filter plate assays according to the manufacturer’s instructions. The relative luminescence was determined.

References

1. Poplutz MK, Wessels I, Rink L, Uciechowski P. Regulation of the Interleukin-6 gene expression during monocytic differentiation of HL-60 cells by chromatin remodeling and methylation. Immunobiology. 2014;219(8):619-26.
