# Supplementary figures and images for: ERK5 modulates IL-6 secretion and contributes to tumor-induced immune suppression
Source: Cell Death Dis. 2021 Oct 20;12(11):969. doi: 10.1038/s41419-021-04257-8 (PMC8528934; doi:10.1038/s41419-021-04257-8)

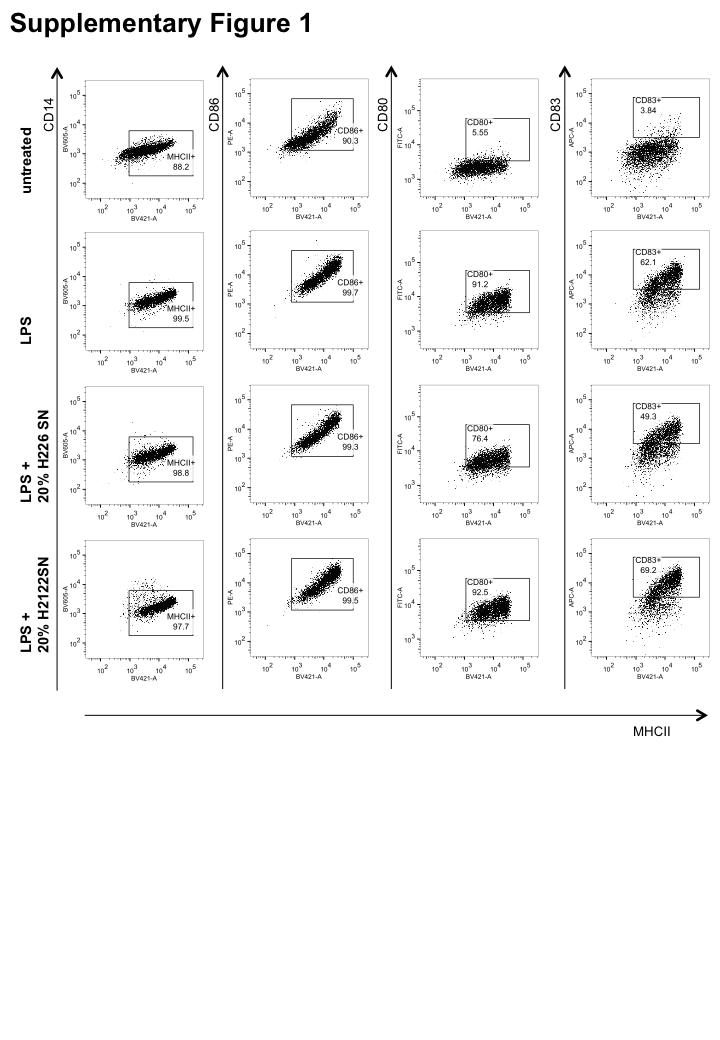

Supplement: Supplementary file 2 — Figure S1 [file 41419_2021_4257_MOESM2_ESM.tif]

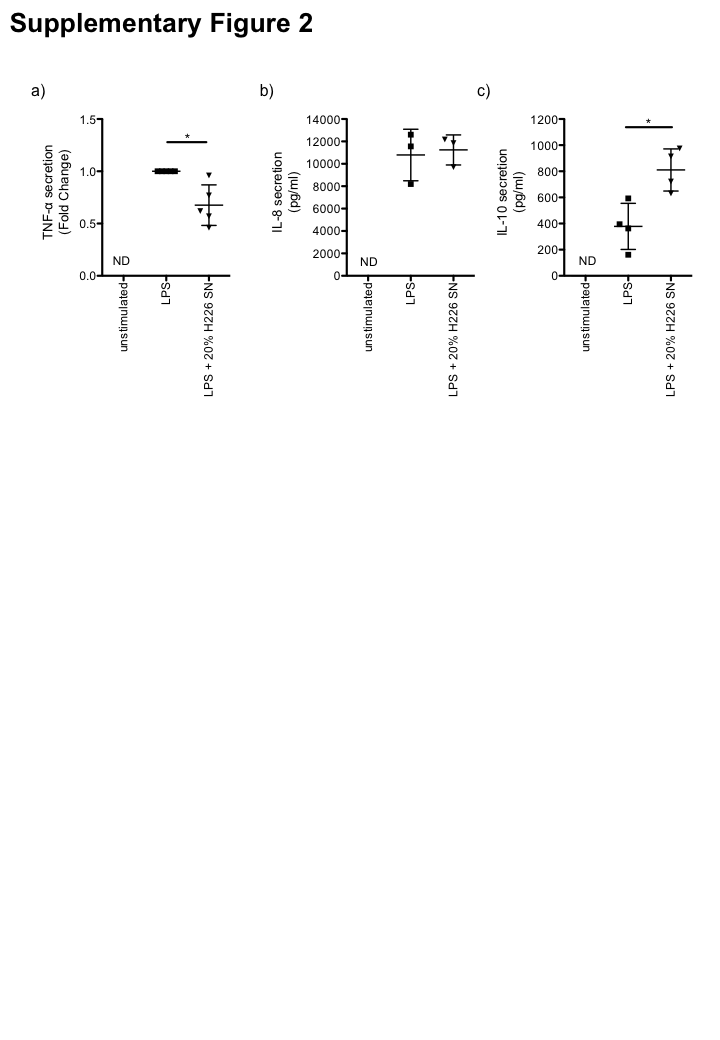

Supplement: Supplementary file 3 — Figure S2 [file 41419_2021_4257_MOESM3_ESM.tif]

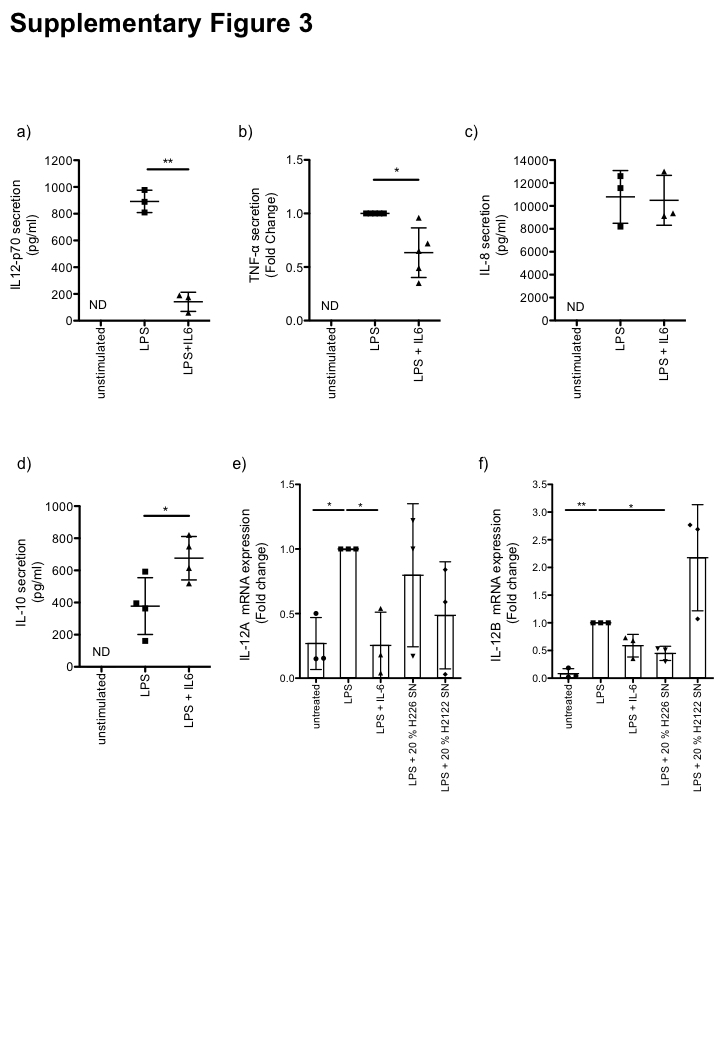

Supplement: Supplementary file 4 — Figure S3 [file 41419_2021_4257_MOESM4_ESM.tif]

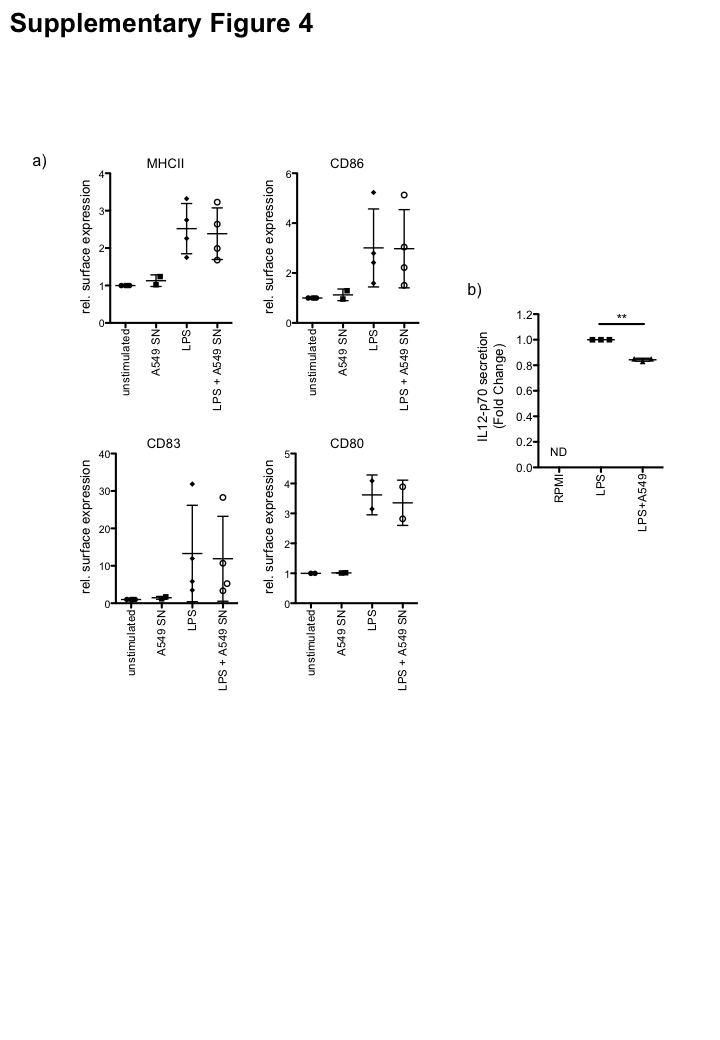

Supplement: Supplementary file 5 — Figure S4 [file 41419_2021_4257_MOESM5_ESM.tif]

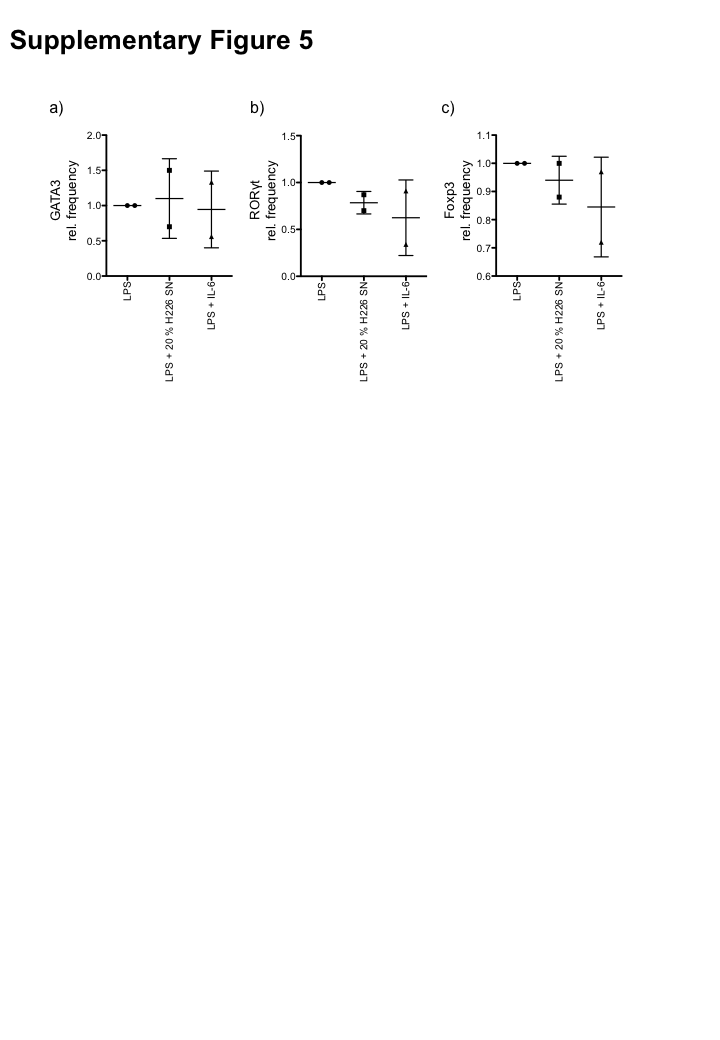

Supplement: Supplementary file 6 — Figure S5 [file 41419_2021_4257_MOESM6_ESM.tif]

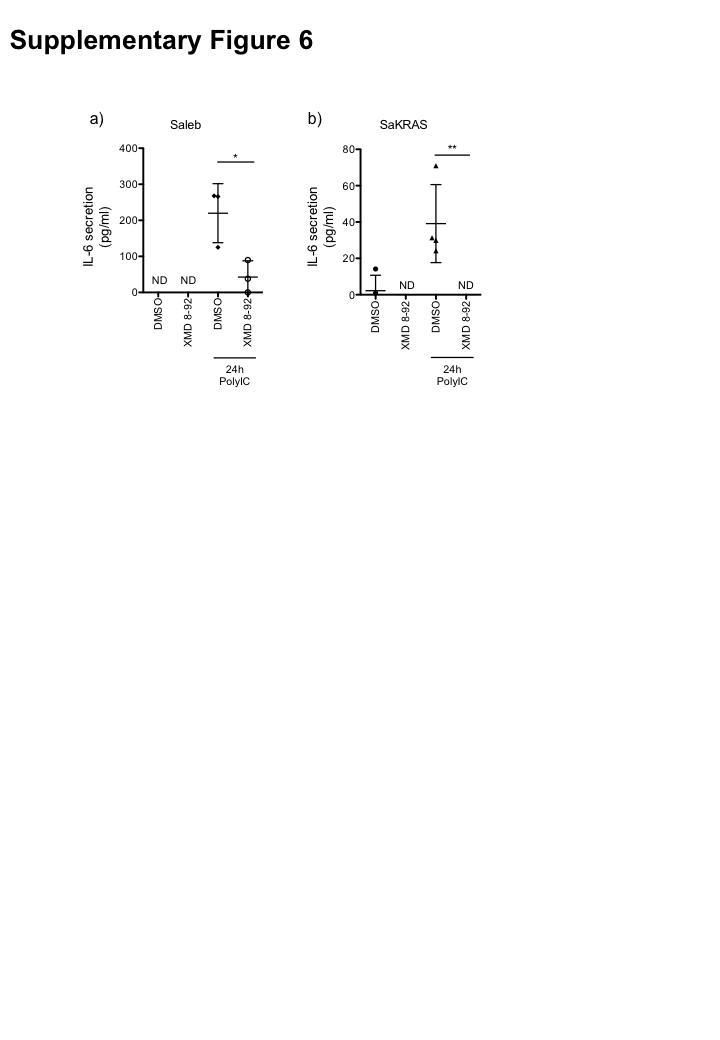

Supplement: Supplementary file 7 — Figure S6 [file 41419_2021_4257_MOESM7_ESM.tif]

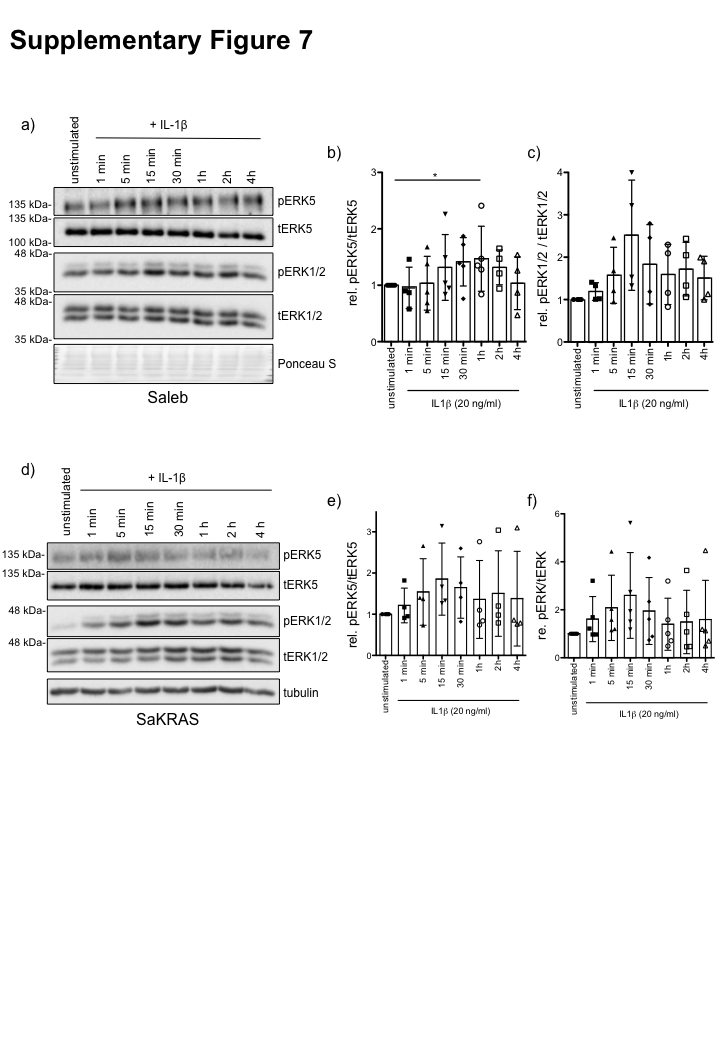

Supplement: Supplementary file 8 — Figure S7 [file 41419_2021_4257_MOESM8_ESM.tif]

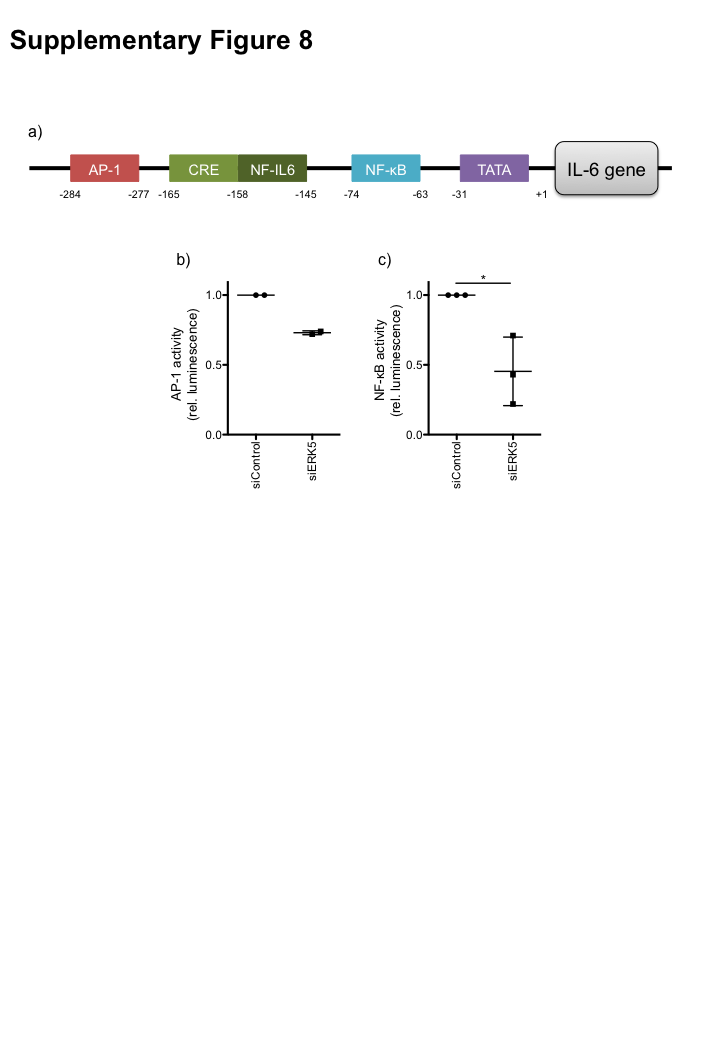

Supplement: Supplementary file 9 — Figure S8 [file 41419_2021_4257_MOESM9_ESM.tif]
